# Neuroprotection in Parkinson’s disease: facts and hopes

**DOI:** 10.1007/s00702-019-02115-8

**Published:** 2019-12-11

**Authors:** András Salamon, Dénes Zádori, László Szpisjak, Péter Klivényi, László Vécsei

**Affiliations:** 1 Department of Neurology, Faculty of Medicine, Interdisciplinary Excellence Centre, Albert Szent-Györgyi Clinical Center, University of Szeged, Semmelweis u. 6., Szeged, 6725 Hungary; 2 MTA-SZTE Neuroscience Research Group, Szeged, Hungary

**Keywords:** Neuroprotection, Parkinson’s disease, Animal models, Clinical trials, Pathomechanism

## Abstract

Parkinson’s disease (PD) is the second most common neurodegenerative disease worldwide. Behind the symptoms there is a complex pathological mechanism which leads to a dopaminergic cell loss in the substantia nigra pars compacta. Despite the strong efforts, curative treatment has not been found yet. To prevent a further cell death, numerous molecules were tested in terms of neuroprotection in preclinical (in vitro, in vivo) and in clinical studies as well. The aim of this review article is to summarize our knowledge about the extensively tested neuroprotective agents (Search period: 1991–2019). We detail the underlying pathological mechanism and summarize the most important results of the completed animal and clinical trials. Although many positive results have been reported in the literature, there is still no evidence that any of them should be used in clinical practice (Cochrane analysis was performed). Therefore, further studies are needed to better understand the pathomechanism of PD and to find the optimal neuroprotective agent(s).

## Introduction

Parkinson’s disease (PD) is the second most common neurodegenerative disease worldwide (Kalia and Lang 2015). Cardinal motor symptoms are bradykinesia, tremor and/or muscle rigidity. Behind the clinical symptoms there is a complex pathological mechanism which leads to dopaminergic cell loss in the substantia nigra pars compacta (Kalia and Kalia 2015). Currently, there is no curative treatment, the gold standard of symptom management is levodopa. At the time of the diagnosis of PD only 30% of the dopaminergic neurons but around 50–60% of their axon terminals have already perished (Cheng et al. 2010). Therefore, in the last few decades great effort has been made to understand the undergoing mechanisms and to find molecule(s) which can protect the dopaminergic neurons from the complex damaging cascade. Our review summarizes the most important pathophysiological aspects of PD and describes the widely studied molecules.

## Review data

The aim of this review article is to provide a comprehensive summary of the pathomechanism and the potential neuroprotective targets in Parkinson’s disease. For search PubMed (MEDLINE) and Web of Science and Cochrane (September 1991 to September 2019) databases were applied. The following search terms were used: ‘Neuroprotection’ AND ‘Parkinson’s disease’ AND ‘6-OHDA’ OR ‘Adenosine A2A’ OR ‘Amantadine’ OR ‘Anti-apoptotic agent’ OR ‘Anti-oxidant’ OR ‘Bromocriptine’ OR ‘Caffeine’ OR ‘Calcium channel antagonist’ OR ‘Coenzyme Q10’ OR ‘CoQ10’ OR ‘Creatine’ OR ‘Deprenyl’ OR ‘Doxycycline’ OR ‘Environment’ OR ‘Exenatide’ OR ‘Exercise’ OR ‘Flavonoid’ OR ‘GDNF’ OR ‘Ghrelin’ OR ‘Isradipine’ OR ‘Kynurenine’ OR ‘Levodopa’ OR ‘MAO inhibitor’ OR ‘Minocycline’ OR ‘MPTP’ OR ‘Neurotropic factors’ OR ‘Neurturin’ OR ‘Nicotine’ OR ‘Nilotinib’ OR ‘NMDA’ OR ‘NSAID’ OR ‘Parkin’ OR ‘Pramipexole’ OR ‘Rasagiline’ OR ‘Rifampicin’ OR ‘Ropinirole’ OR ‘Selegiline’ OR ‘Tocopherol’ OR ‘UCH-L1’ OR ‘Uric acid’ OR ‘Uridine’ OR ‘Vitamin D’ OR ‘Vitamin E’ OR ‘α-synuclein’ OR ‘Vitamin C’ AND ‘Review’. The most comprehensive, online available reviews have been selected for further evaluation and after the collection of all necessary information they were synthesized in this article.

## Pathogenesis of Parkinson’s disease

**Fig. 1:**
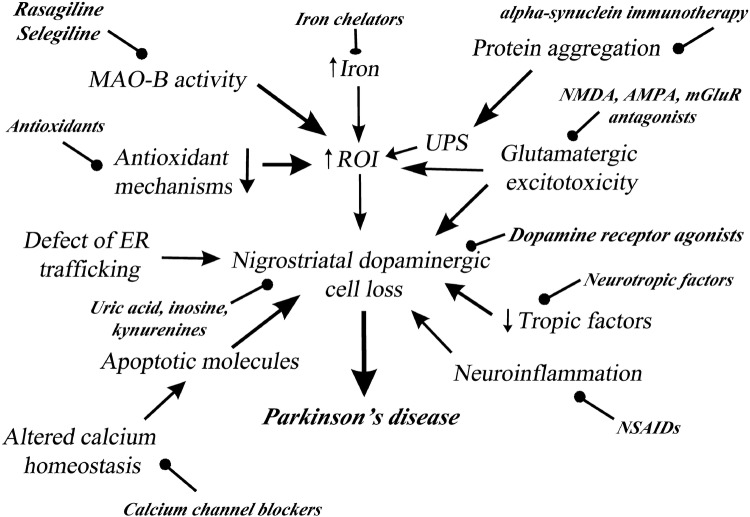
Patomechanism and potential neuroprotective targets in Parkinson’s disease (*AMPA* α-amino-3-hydroxy-5-methyl-4-isoxazolepropionic acid receptor, *ER* endoplasmatic reticulum, *MAO*-*B* monoamine oxidase B, *mGluR* metabotropic glutamatergic receptor, *NMDA* N-methyl-D-aspartate receptor, *NSAID* non-steroidal anti-inflammatory drug, *ROI* reactive oxygen intermediates, *UPS* ubiquitin–proteasome system)

Despite extensive animal and clinical studies, the etiology of PD is still unclear (Fig. 1). Most of our information on the pathomechanism of Parkinson’s disease originate from 1-methyl-4-phenyl-1,2,3,6-tetrahydropyridine (MPTP) and 6-hydroxydopamine (6-OHDA) animal models. Presumably PD is much more complex than can be modeled with toxin experiments. The most important pathological processes are the followings (Mandel et al. 2003; Allain et al. 2008):

(1) Monoamine oxidase B (MAO-B) activity—it is widely known that MAO-B metabolizes the MPTP toxin to its active compound, 1-methyl-4-phenylpyridium (MPP^+^), which reaction creates reactive oxygen intermediates (ROI) and lead to cell death (Mandel et al. 2003). It could be one reason why selegiline is effective in MPTP toxin models (Heikkila et al. 1984). In PD there is an accumulation of alpha-synuclein aggregates in the astrocytes. This accumulation results in oxidative stress. A previously published research reported that there is a positive correlation between MAO-B and astrocyte marker levels (e.g., glial fibrillary acidic protein). Therefore, it seems that MAO-B plays an important pathogenic role in the production of ROI in the activated astrocytes (Jellinger 2017; Langston 2017; Tong et al. 2017). (2) Oxidative stress and reduced endogenous antioxidant capacity (Zádori et al. 2011). (3) Elevated iron level—It is hypothesized that oxidative stress, which is provoked by iron metabolism, is one of the most important cause of neurodegeneration (Mandel et al. 2003). (4) Glutamatergic excitotoxicity (Koutsilieri and Riederer 2007; Majláth et al. 2016a; Zádori et al. 2012a, 2013). (5) Abnormal protein aggregation, misfolding—Parkinson’s disease is a sporadic disease. However, rarely familial (estimated incidence 1–2%) forms were also reported in the scientific literature (Polymeropoulos et al. 1997). If there is a mutation in the *SNCA* gene, α-synuclein starts to aggregate and it seems that this aggregated protein interferes with ubiquitin–proteasome system (Chung et al. 2001). The production of Lewy bodies is accelerated by the aggregation of the α-synuclein forming proteins. Currently around 20 genes have been identified (Kim and Alcalay 2017). (6) Reduced level of trophic factors (see in “Neurotropic factors”). (7) Altered ion (calcium) homeostasis (Hirsch et al. 2013). (8) Neuroinflammation—it has been showed that cyclooxygenase (COX) COX-2 is upregulated in Parkinsonian patients. The pharmacological inhibition of this enzyme leads to the prevention of toxic dopamine-quinone formation in MPTP mouse model (Teismann et al. 2003). Theoretically microglias may contribute to the ongoing cell death by producing inflammatory molecules, such as prostaglandins, interleukins and reactive oxygen species (Allain et al. 2008). (9) Apoptosis—in animal studies and also in Parkinsonian patients there is an upregulation of the synthesis of proteins which take part in the apoptotic pathways. P53, caspase-3 are just two of the many involved proteins (Allain et al. 2008; Stern 1996). (10) Defect of the endoplasmatic reticulum trafficking system—in the normal cells, α-synuclein contributes to the synaptic vesicle recycling and to the maintenance of the membrane plasticity (Bonini and Giasson 2005). Nonetheless, the aggregation of these proteins leads to a lethal block in the vesicular transport mechanisms (endoplasmatic reticulum, Golgi) (Allain et al. 2008).

## Neuroprotective agents

Neuroprotection is mostly a pharmacological intervention that slows the natural progression of the PD or helps to save the most vulnerable dopaminergic neurons in the substantia nigra. This section summarizes the main animal and clinical experimental results of the compounds tested for neuroprotection in PD.

### MAO-B inhibitors

Selegiline (Tábi et al. 2019) is used in the daily practice to manage on/off fluctuations and to reduce the levodopa dose (Lees et al. 1977). Selegiline reduces the oxidative stress, which is produced by the metabolism of biogenic amines and environmental toxic agents (e.g., pesticides). It elevates the endogenous anti-oxidant capacity (superoxide dismutase (SOD) and catalase) and prevents the uptake of neurotoxins in the nerve terminals (Mandel et al. 2003). Two important prospective, double-blind trials (DATATOP and SINDEPAR) were performed (in this review we do not summarize the clinical trials of Tetrud and Langston (Tetrud and Langston, 1989), Swedish—(Pålhagen et al. 1998) and Norwegian-Danish (Larsen et al. 1999) selegiline trials, because the small number of participants and the limited amount of available data). The DATATOP study assessed the effect of selegiline and tocopherol on the progression of PD. Untreated patients were randomly separated in different groups: (1) vitamin E (2000 IU); (2) selegiline (deprenyl) (2 × 5 mg/day); (3) vitamin E and selegiline (deprenyl); (4) placebo. In the vitamin E group, there was no observable benefit, but in the selegiline groups there was a delay in the time needed to start the levodopa treatment compared to placebo. It could not be decided whether the drug had a neuroprotective or just a prolonged symptomatic effect. The aim of the SINDEPAR (Sinemet-Deprenyl-Parlodel) study was to estimate the potential neuroprotective effect of selegiline in untreated patients. The primary endpoint was the change in the Unified Parkinson’s Disease Rating Scale (UPDRS) score between first and last visits (14 months (12 treatment + 2 months washout period). Although the selegiline group has lesser worsening on the UPDRS scale, it was hard to exclude the potential long-term symptomatic effect of selegiline (Parkinson study group 1989, 1993; Olanow et al. 1995; Olanow and Jankovic 2005).

Rasagiline is a more potent MAO-B inhibitor than selegiline, without amphetamine-like metabolites (Youdim et al. 2001; Youdim 2010; Weinreb et al. 2011). Animal and cell culture studies concluded that rasagiline increases the antioxidant capacity (SOD, catalase, bcl-2) and prevents toxic effects of peroxynitrite, MPTP and 6-OHDA. Rasagiline increases the survival rate of dopaminergic neurons as well. In clinical phase rasagiline (up to 2 mg/day) as an adjuvant to levodopa was examined during 12 weeks of treatment. There was a persistent improvement in the UPDRS scores compared to placebo (Rabey et al. 2000). Furthermore, TEMPO trial found that early treatment with rasagiline slowed down the progression of the symptoms compared to the delayed started (6 month later) group (Parkinson Study Group 2002). Olanow et al. performed a similar clinical trial (ADAGIO; 1176 subjects). They found that 1 mg rasagiline lead to a significant improvement in the UPDRS score, while 2 mg did not reach the significance (Olanow et al. 2009). In the PRESTO study 472 Parkinsonian patients were involved with at least 2 ½ h daily OFF-time. They found an improvement in the UPDRS and in the global impression scale after rasagiline administration. 29% OFF-time reduction was detectable in the 1.0 mg/day rasagiline group (Parkinson Study Group 2005). In the LARGO trial during 18-week 687 Parkinsonian patients were randomly divided in rasagiline (1 mg/day), entacapone (200 mg with every levodopa) and placebo groups. In the rasagiline group there was a decrease in the daily OFF-time (− 1.18 h) and an increase in the ON-time without troublesome dyskinesias (Rascol et al. 2005). These trials did not make easier the interpretation of the potential neuroprotective effect of rasagiline. Peretz et al. published in (2016) a real-life study, where no differences were found on the natural PD history between patients, who were treated with rasagiline or selegiline.

### Dopamine receptor agonists

Dopamine receptor agonists may be able to prevent the nigrostriatal dopaminergic cell loss, perhaps as a consequence of antioxidant and levodopa sparing effects (Djaldetti and Melamed 2002). These compounds stimulate the dopaminergic auto-receptors as well, resulting in a decrease in dopamine excretion (Djaldetti and Melamed 2002).

In animal studies, pramipexole (*D*2/*D*3 receptor agonist), bromocriptine (*D*2 receptor agonist), R-apomorphine (*D*1/*D*2 receptor agonist), ropinirole (*D*2/*D*3 receptor agonist) were tested in different models (ischemic damage, methamphetamine, 3,4-dihydroxyphenylacetic acid and homovanillic acid depletion, MPTP, 6-OHDA). The applied dopamine agonists prevented the loss of dopaminergic neurons (Fornai et al. 2001; Grünblatt et al. 1999; Hall et al. 1996; Iida et al. 1999; Kondo et al. 1994; Zou et al. 2000).

In clinical studies, it is an unsolved question whether these compounds have a neuroprotective or just a symptomatic effect (Djaldetti and Melamed 2002). PET and SPECT studies were performed with the aim of better distinction between these 2 kinds of effects (Djaldetti and Melamed 2002). In the REAL PET study ropinirole was compared to levodopa (Whone et al. 2003) and the nigrostriatal system fluorodopa uptake was measured. This uptake was slower in the ropinirole group, but no correlation between the neuroimaging findings and the patient’s clinical status was found there. A similar study (CALM-PD) was conducted with pramipexole, where β-CIT labeled with ^[123]^iodine striatal uptake (SPECT) was compared to the levodopa group (Marek et al. 2002). Although the study duration was short (46 months), there was a clear positive tendency in the pramipexole group (Marek et al. 2002). This result was not confirmed in the PROUD study (Schapira 2013). None of these neuroimaging studies had placebo control groups. Therefore, it is hard to decide which of the following two interpretations of the results is the correct one: (1) dopamine receptor agonists have neuroprotective effect or (2) levodopa induces the degeneration of dopaminergic neurons (Olanow and Jankovic 2005). In the ELLDOPA trial, untreated patients were involved and randomly divided in the following groups: 300–450–600 mg levodopa/day or placebo. There was a lower clinical decline in levodopa groups compared to placebo. It demonstrated the potential protective effect of levodopa treatment (The Parkinson Study Group 2004). On the contrary, a study published in 2019 found, that in a delay-start clinical trial (445 patients; 80 week levodopa treatment (3 × 100 mg levodopa + 25 mg carbidopa) vs. 40 week placebo followed by 40 week levodopa treatment) there was no detectable disease-modifying effect of levodopa (Verschuur et al. 2019).

### NMDA receptor antagonists

Next to the dopaminergic system, alterations in glutamatergic transmission also contributes to the development of the Parkinsonian symptoms via excitotoxicity (Mandel et al. 2003). Riluzole exerted protective effect on dopaminergic cells in the MPTP toxin model. In the clinical phase no significant alteration occurred in the UPDRS score of riluzole treated groups; therefore, the clinical trial was interrupted (Obinu et al. 2002; Jankovic and Hunter 2002). Amantadine has a mild retarding effect on the progression of the disease in MPTP model. In the clinical use, amantadine has a good clinical effect on the motor complications (Rojas et al. 1992; Schwab et al. 1972; Greulich and Fenger 1995). The retrospective analysis of patients treated with amantadine showed an improved survival rate (Uitti et al. 1996). Memantine exerted a dose-dependent, sustained effect on cortical and hippocampal neurons induced by excitotoxicity and hypoperfusion (Seif el Nasr et al. 1990; Erdö and Schäfer 1991). NMDA-receptor antagonists are badly tolerated by patients because of their side effects (e.g., psychiatric symptoms) (Olanow and Jankovic 2005).

### Iron chelators

Nigral iron deposition, located mainly in the glia cells, is characteristic of PD as well. Iron promotes the development of oxidative stress which leads to protein misfolding and the formation of Lewy bodies (Sian-Hülsmann et al. 2010). Despite the clear evidence on iron chelators [e.g., deferoxamine, phytic acid (IP6)] in the preclinical models, there is no data on their clinical utility (Gerlach et al. 1994; Mochizuki et al. 1994; Dusek et al. 2016; Seidl and Potashkin 2011).

### Neurotropic factors

In animal models the effect of the growth factors was widely studied (Djaldetti and Melamed 2002). It was identified in the MPTP-treated monkeys that glial derived neurotropic factor (GDNF) helped to return the dopaminergic cell function (Djaldetti and Melamed 2002). MPTP treated monkeys were infused with GDNF (intraventricular or striatal) 3 months after lesioning. There was an obvious increase in the number and the size of the tyrosine hydroxylase (TH)-positive cells (Schapira 2008). Similar results were detectable with lentiviral transfectioning after 1 week of MPTP treatment (Kordower et al. 2000). GDNF did not penetrate adequately to the target after intraventricular administration (Kordower et al. 1999). In the first phase II study, GDNF was administered intracerebroventricularly without any benefit (Domanskyi et al. 2015; Nutt et al. 2003a, b). In view of the safety of the intraputaminal administration in one study, this route of administration was used in the second clinical phase II trial. This treatment did not result in clinical improvement (Lang et al. 2006). Concomitant administration of GDNF in putamen and substantia nigra was also tested with good safety in 6 PD patients (Bartus et al. 2013).

### Calcium channel blockers

There is no clear evidence on the effectiveness of calcium channel blockers in Parkinson’s disease (Seidl and Potashkin 2011). Isradipine (L-type dihydropyridine) was successful in the prevention of the apoptosis after hypoxic damage (Barhwal et al. 2009). The L-type channel blockers seem to be protective after MPTP or 6-OHDA administration in animals (Bove et al. 2005; Kalia et al. 2015). In a clinical phase (STEADY-PD—multicenter, phase II study) isradipine was tested on 99 early Parkinsonian subjects (5, 10 or 20 mg/day or placebo). They found that the maximal tolerable dose is 10 mg/day (Parkinson Study Group 2013). Isradipine is under testing in an ongoing phase III clinical trial (NCT02168842).

### Coenzyme Q10

Coenzyme Q10 (Co-Q10; ubiquinone) functions as an antioxidant and as a part of the respiratory chain of mitochondria. It has the capacity to scavenge free radicals. In a pilot study, patients were randomly divided into placebo and treated groups [3 doses (300 mg/d; 600 mg/d; 1200 mg/d)]. Patients who got the highest Co-Q10 dose (1200 mg/d) had a short-term improvement and a significant lower deterioration in the UPDRS score compared to placebo group (Shults et al. 2002). A small trial was performed, where Parkinsonian patients were treated with 360 mg Co-Q10 daily over 4 weeks (Müller et al. 2003). It showed a mild symptomatic effect after the treatment. Larger study did not find any effect on the motor symptoms of PD after the administration of 300 mg Co-Q10 daily (Storch et al. 2007). Another randomized phase III clinical trial found no benefit after 1200 mg/day or 2400 mg/day Co-Q10 administration (+ vitamin E 1200 IU/day was concomitantly administered) (The Parkinson Study Group QE3 Investigators 2014).

### Creatine

Creatine is metabolized to phosphocreatine in the cell. This phosphate group could help in the stage of energy deprivation across the synthesis of ATP from ADP. In the MPTP animal model creatine had a protective effect on dopaminergic cell loss (Matthews et al. 1999). In the NINDS NET-PD study (2006) 10 g creatine was administered daily to 66 early Parkinsonian patients. The clinical trial was terminated early because of the futility of results (The NINDS-PD Investigators 2006). In another study 2 g creatine was used for 6 months, then 4 g for 18 months in 31 patients. There was no significant change either in the UPDRS score, or in the SPECT results compared to the placebo group (Bender et al. 2006).

### Alpha-synuclein immunotherapy

There are hypotheses in the literature, that prion-like mechanisms play important role in the development of PD (Visanji et al. 2013). These treatments targeting the extracellular toxic form of the α-synuclein. PD01A is a short peptide, which contains the C-terminus of the alpha-synuclein. In a phase I trial, 24 early Parkinsonian patients were involved to test this compound. It was safe and well tolerated after 12 months of treatment period with two subcutaneously administered different doses. PD03A, another compound was also tested in a phase I randomized controlled trial (RCT). Currently, there are no available results (NCT02267434). Not only active immunization therapies were examined, but even the passive ones as well. PRX002 is a monoclonal anti-α-synuclein antibody, which was safe and well tolerated (single and multiple doses). Currently there is a phase II clinical study ongoing testing its efficacy (NCT03100149) (Jankovic et al. 2018). Further clinical trials are ongoing or under planning with monoclonal antibodies (e.g., BIIB054, BAN0805), α-synuclein aggregation modulators (e.g., NPT200-11, − 088, ANLE 138b) and Glucocerebrosidase enhancers (e.g., Ambroxol, GZ/SAR4027671) (Fernández-Valle et al. 2019). To reach a higher efficacy in the prevention of synuclein accumulation Rockenstein et al. tested and reported the positive effect of the combination of humoral and cellular immunization (glucan microparticle (GP) + rapamycin (RAP)/α-syn) in PDGF-α-syn transgenic mice (Rockenstein et al. 2018). Spencer et al. reported that short interfering RNA oligonucleotides (siRNA) could be an interesting new tool in the treatment of synucleinopathies. They identified an alternative peptide [apolipoprotein B (ApoB)] which allows the transport of oligonucleotides into the nervous system across the blood brain barrier. This method showed efficacy and revealed reduction of the neuropathological alteration severity in transgenic animal model of Parkinson’s disease (Spencer et al. 2019).

### Recreational—caffeine, nicotine

The mount of evidence supports the possible neuroprotective effect of caffeine (Ross et al. 2000; Ascherio et al. 2001; Saaksjarvi et al. 2008). Acute and chronic administration of caffeine reduced the striatal dopamine cell loss in the MPTP, 6-OHDA and paraquat + maneb animal models (Chen et al. 2001; Joghataie et al. 2004; Kachroo et al. 2010). A pilot study was also performed in Parkinsonian patients who received 200–400 mg/day caffeine for 6 weeks. The improvement of motor symptoms was observed on the UPDRS score (NCT01738178). Caffeine is an adenosine A_2A_ receptor antagonist (Prediger 2010; Seidl and Potashkin 2011). Another adenosine A_2A_ receptor blockers are in a different stage of the drug developmental process (e.g., Istradefylline, Preladenant, V81444, tozadenant, ST1535, PBF-509, ST4203) (Bara-Jimenez et al. 2003; Cutler et al. 2012; Tarazi et al. 2014).

In a large epidemiological Chinese study, there was a lower incidence of PD in that part of the population who regularly drink coffee and smoke cigarettes (Tan et al. 2003). Nicotine use without any caffeine consumption also has a risk reducing effect (Qick 2004; Godwin-Austen et al. 1982). The neuroprotective effect of nicotine in animal studies was eliminable after administration of nicotine receptor antagonist. This suggests that neuroprotective effect of nicotine is mediated by the cerebral nicotinic cholinergic receptors (nAChR) (Quik and Jeyarasasingam 2000). Currently there is a phase II clinical trial ongoing (transdermal nicotine) (NIC-PD) (NCT01560754).

### Uric acid, Inosine

Interestingly, slower decline was detectable in clinical symptoms of PD in the population with higher blood level of uric acid (Ascherio et al. 2009; De Lau et al. 2005). Animal studies reported that uric acid prevents dopaminergic cell death via antioxidant mechanisms (Duan et al. 2002). The conclusion of these studies is that in parkinsonian patients the elevated uric acid level is not always needed to be treated (Seidl and Potashkin 2011). Inosine is a precursor molecule of urate. In a phase II RCT study (SURE-PD) inosine was tested in 75 early Parkinsonian patients. The treatment for 8–24 months elevated the serum and the cerebrospinal fluid urate content (Schwarzschild 2014).

### Kynurenines

Numerous data support the role of kynurenines in neurological diseases, including PD (Bohár et al. 2015; Majláth et al. 2016b; Klivényi et al. 2004; Vécsei et al. 2013; Zádori et al. 2012b). One of the most important compounds of this system is kynurenic acid (KYNA) (Sas et al. 2007). In vitro and also in vivo (animal) experiments demonstrated protective effect of KYNA after toxin administration (e.g., MPTP, QUIN). The most important challenge in this field is to find the solution for its short elimination time and its low penetration through the blood–brain-barrier (Zádori et al. 2011).

## Conclusion

Although many molecules have been extensively tested in preclinical (in vitro, in vivo) and clinical studies, no perfect drug was found. Most of our knowledge comes from toxin animal models, allowing us to study only one part of the pathological mechanism. Some authors hypothesized that the combination of the tested neuroprotective agents (‘coctail’) could be effective. Currently there is a wide-range of studies ongoing with molecules (e.g., antidiabetics (exenatide), anticancer drugs (nilotinib), glutamate (AMPA; metabotropic) receptor antagonists (LY-300164, perampanel, talampanel, AFQ056, dipraglurant), neurotrophic factors (cerebral dopamine and astrocyte-derived neurotropic factors (CDNF and MANF), melatonin, acyl-ghrelin mimetics). We think that besides further development of chemical molecules the identification of novel molecular drug targets is also needed.

## References

[CR1] Allain H, Bentué-Ferrer D, Akwa Y (2008). Disease-modifying drugs and Parkinson’s disease. Prog Neurobiol.

[CR2] Ascherio A, Zhang SM, Hernan MA (2001). Prospective study of caffeine consumption and risk of Parkinson’s disease in men and women. Ann Neurol.

[CR3] Ascherio A, LeWitt PA, Xu K (2009). Urate as a predictor of the rate of clinical decline in Parkinson disease. Arch Neurol.

[CR4] Bara-Jimenez W, Sherzai A, Dimitrova T (2003). Adenosine A(2A) receptor antagonist treatment of Parkinson’s disease. Neurology.

[CR5] Barhwal K, Hota SK, Baitharu I (2009). Isradipine antagonizes hypobaric hypoxia induced CA1 damage and memory impairment: complementary roles of L-type calcium channel and NMDA receptors. Neurobiol Dis.

[CR6] Bartus RT, Baumann TL, Siffert J (2013). Safety/feasibility of targeting the substantia nigra with AAV2-neurturin in Parkinson patients. Neurology.

[CR7] Bender A, Koch W, Elstner M (2006). Creatine supplementation in Parkinson disease: a placebo-controlled randomized pilot trial. Neurology.

[CR8] Bohár Z, Toldi J, Fülöp F (2015). Changing the face of kynurenines and neurotoxicity: therapeutic considerations. Int J Mol Sci.

[CR9] Bonini NM, Giasson BI (2005). Snaring the function of alpha-synuclein. Cell.

[CR10] Bove J, Prou D, Perier C (2005). Toxin-induced models of Parkinson’s disease. NeuroRx.

[CR11] Chen JF, Xu K, Petzer JP (2001). Neuroprotection by caffeine and A(2A) adenosine receptor inactivation in a model of Parkinson’s disease. J Neurosci.

[CR12] Cheng HC, Ulane CM, Burke RE (2010). Clinical progression in Parkinson disease and the neurobiology of axons. Ann Neurol.

[CR13] Chung KK, Dawson VL, Dawson TM (2001). The role of the ubiquitin-proteasomal pathway in Parkinson’s disease and other neurodegenerative disorders. Trends Neurosci.

[CR14] Cutler DL, Tendolkar A, Grachev ID (2012). Safety, tolerability and pharmacokinetics after single and multiple doses of preladenant (SCH420814) administered in healthy subjects. J Clin Pharm Ther.

[CR15] De Lau LM, Koudstaal PJ, Hofman A (2005). Serum uric acid levels and the risk of Parkinson disease. Ann Neurol.

[CR16] Djaldetti R, Melamed E (2002). New drugs in the future treatment of Parkinson’s disease. J Neurol.

[CR17] Domanskyi A, Saarma M, Airavaara M (2015). Prospects of neurotrophic factors for Parkinson’s disease: comparison of protein and gene therapy. Hum Gene Ther.

[CR18] Duan W, Ladenheim B, Cutler RG (2002). Dietary folate deficiency and elevated homocysteine levels endanger dopaminergic neurons in models of Parkinson’s disease. J Neurochem.

[CR19] Dusek P, Schneider SA, Aaseth J (2016). Iron chelation in the treatment of neurodegenerative diseases. J Trace Elem Med Biol.

[CR20] Erdö SL, Schäfer M (1991). Memantine is highly potent in protecting cortical cultures against excitotoxic cell death evoked by glutamate and N-methyl-D-aspartate. Eur J Pharmacol.

[CR21] Fernández-Valle T, Gabilondo I, Gómez-Esteban JC (2019). New therapeutic approaches to target alpha-synuclein in Parkinson’s disease: The role of immunotherapy. Int Rev Neurobiol.

[CR22] Fornai F, Battaglia G, Gesi M (2001). Dose-dependent protective effects of apomorphine against methamphetamine-induced nigrostriatal damage. Brain Res.

[CR23] Gerlach M, Ben-Shachar D, Riederer P (1994). Altered brain metabolism of iron as a cause of neurodegenerative diseases?. J Neurochem.

[CR24] Godwin-Austen RB, Lee PN, Marmot MG (1982). Smoking and Parkinson’s disease. J Neurol Neurosurg Psychiatry.

[CR25] Greulich W, Fenger E (1995). Amantadine in Parkinson’s disease: pro and contra. J Neural Transm Suppl.

[CR26] Grünblatt E, Mandel S, Berkuzki T (1999). Apomorphine protects against MPTP-induced neurotoxicity in mice. Mov Disord.

[CR27] Hall ED, Andrus PK, Oostveen JA (1996). Neuroprotective effects of the dopamine D2/D3 agonist pramipexole against postischemic or methamphetamine-induced degeneration of nigrostriatal neurons. Brain Res.

[CR28] Heikkila RE, Manzino L, Cabbat FS (1984). Protection against the dopaminergic neurotoxicity of 1-methyl-4-phenyl-1,2,5,6-tetrahydropyridine by monoamine oxidase inhibitors. Nature.

[CR29] Hirsch EC, Jenner P, Przedborski S (2013). Pathogenesis of Parkinson’s disease. Mov Disord.

[CR30] Iida M, Miyazaki I, Tanaka K (1999). Dopamine D2 receptor-mediated antioxidant and neuroprotective effects of ropinirole, a dopamine agonist. Brain Res.

[CR31] Jankovic J, Hunter C (2002). A double-blind, placebo-controlled and longitudinal study of riluzole in early Parkinson’s disease. Parkinsonism Relat Disord.

[CR32] Jankovic J, Goodman I, Safirstein B (2018). Safety and tolerability of multiple ascending doses of PRX002/RG7935, an anti-α-synuclein monoclonal antibody, in patients with Parkinson disease: a randomized clinical trial. JAMA Neurol.

[CR33] Jellinger KA (2017). Brain monoamine oxidases in human parkinsonian disorders. Brain.

[CR34] Joghataie MT, Roghani M, Negahdar F (2004). Protective effect of caffeine against neurodegeneration in a model of Parkinson’s disease in rat: behavioral and histochemical evidence. Parkinsonism Relat Disord.

[CR35] Kachroo A, Irizarry MC, Schwarzschild MA (2010). Caffeine protects against combined paraquat and maneb-induced dopaminergic neuron degeneration. Exp Neurol.

[CR36] Kalia LV, Kalia SK (2015). α-Synuclein and Lewy pathology in Parkinson’s disease. Curr Opin Neurol.

[CR37] Kalia LV, Lang AE (2015). Parkinson’s disease. Lancet.

[CR38] Kalia LV, Kalia SK, Lang AE (2015). Disease-modifying strategies for Parkinson’s disease. Mov Disord.

[CR39] Kim CY, Alcalay RN (2017). Genetic forms of Parkinson’s disease. Semin Neurol.

[CR40] Klivényi P, Toldi J, Vécsei L (2004). Kynurenines in neurodegenerative disorders: therapeutic consideration. Adv Exp Med Biol.

[CR41] Kondo T, Ito T, Sugita Y (1994). Bromocriptine scavenges methamphetamine-induced hydroxyl radicals and attenuates dopamine depletion in mouse striatum. Ann N Y Acad Sci.

[CR42] Kordower JH, Palfi S, Chen EY (1999). Clinicopathological findings following intraventricular glial-derived neurotrophic factor treatment in a patient with Parkinson’s disease. Ann Neurol.

[CR43] Kordower JH, Emborg ME, Bloch J (2000). Neurodegeneration prevented by lentiviral vector delivery of GDNF in primate models of Parkinson’s disease. Science.

[CR44] Koutsilieri E, Riederer P (2007). Excitotoxicity and new antiglutamatergic strategies in Parkinson’s disease and Alzheimer’s disease. Parkinsonism Relat Disord.

[CR45] Lang AE, Gill S, Patel NK (2006). Randomized controlled trial of intraputamenal glial cell line-derived neurotrophic factor infusion in Parkinson disease. Ann Neurol.

[CR46] Langston JW (2017). The MPTP story. J Parkinsons Dis.

[CR47] Larsen JP, Boas J, Erdal JE (1999). Does selegiline modify the progression of early Parkinson’s disease? Results from a five-year study. The Norwegian-Danish Study Group. Eur J Neurol.

[CR48] Lees AJ, Shaw KM, Kohout LJ (1977). Deprenyl in Parkinson’s disease. Lancet.

[CR49] Majláth Z, Toldi J, Fülöp F (2016). Excitotoxic mechanisms in non-motor dysfunctions and levodopa- induced dyskinesia in Parkinson’s disease: the role of the interaction between the dopaminergic and the kynurenine system. Curr Med Chem.

[CR50] Majláth Z, Török N, Toldi J (2016). Memantine and kynurenic acid: current neuropharmacological aspects. Curr Neuropharmacol.

[CR51] Mandel S, Grünblatt E, Riederer P (2003). Neuroprotective strategies in Parkinson’s disease: an update on progress. CNS Drugs.

[CR52] Marek K, Seibyl J, Shoulson I (2002). Dopamine transporter brain imaging to assess the effects of pramipexole vs levodopa on Parkinson disease progression. JAMA.

[CR53] Matthews RT, Ferrante RJ, Klivenyi P (1999). Creatine and cyclocreatine attenuate MPTP neurotoxicity. Exp Neurol.

[CR54] Mochizuki H, Imai H, Endo K (1994). Iron accumulation in the substantia nigra of 1-methyl-4-phenyl-1,2,3,6-tetrahydropyridine (MPTP)-induced hemiparkinsonian monkeys. Neurosci Lett.

[CR55] Müller T, Büttner T, Gholipour AF (2003). Coenzyme Q10 supplementation provides mild symptomatic benefit in patients with Parkinson’s disease. Neurosci Lett.

[CR56] NINDS NET-PD Investigators (2006). A randomized, double-blind, futility clinical trial of creatine and minocycline in early Parkinson disease. Neurology.

[CR57] Nutt JG, Burchiel KJ, Comella CL (2003). Implanted intracerebroventricular. Glial cell line-derived neurotrophic factor. Randomized, double-blind trial of glial cell line-derived neurotrophic factor (GDNF) in PD. Neurology.

[CR58] Nutt JG, Burchiel KJ, Comella CL (2003). Randomized, double-blind trial of glial cell line-derived neurotrophic factor (GDNF) in PD. Neurology.

[CR59] Obinu MC, Reibaud M, Blanchard V (2002). Neuroprotective effect of riluzole in a primate model of Parkinson’s disease: behavioral and histological evidence. Mov Disord.

[CR60] Olanow CW, Jankovic J (2005). Neuroprotective therapy in Parkinson’s disease and motor complications: a search for a pathogenesis-targeted, disease-modifying strategy. Mov Disord.

[CR61] Olanow CW, Hauser RA, Gauger L (1995). The effect of deprenyl and levodopa on the progression of signs and symptoms in Parkinson’s disease. Ann Neurol.

[CR62] Olanow CW, Rascol O, Hauser R (2009). A double-blind, delayed-start trial of rasagiline in Parkinson’s disease. N Engl J Med.

[CR63] Pålhagen S, Heinonen EH, Hägglund J (1998). Selegiline delays the onset of disability in de novo parkinsonian patients. Swedish Parkinson Study Group. Neurology.

[CR64] Parkinson Study Group (1989). DATATOP: a multicenter controlled clinical trial in early Parkinson’s disease. Arch Neurol.

[CR65] Parkinson Study Group (1993). Effects of tocopherol and Deprenyl on the progression of disability in early Parkinson’s disease. N Eng J Med.

[CR66] Parkinson Study Group (2002). A controlled trial of rasagiline in early Parkinson disease: the TEMPO Study. Arch Neurol.

[CR67] Parkinson Study Group (2005). A randomized placebo-controlled trial of rasagiline in levodopa-treated patients with Parkinson disease and motor fluctuations: the PRESTO study. Arch Neurol.

[CR68] Parkinson Study Group (2013). Phase II safety, tolerability, and dose selection study of isradipine as a potential disease-modifying intervention in early Parkinson’s disease (STEADY-PD). Mov Disord.

[CR69] Schwarzschild MA, Ascherio A, Parkinson Study Group SURE-PD Investigators (2014). Inosine to increase serum and cerebrospinal fluid urate in Parkinson disease: a randomized clinical trial. JAMA Neurol.

[CR70] Peretz C, Segev H, Rozani V (2016). Comparison of selegiline and rasagiline therapies in parkinson disease: a real-life study. Clin Neuropharmacol.

[CR71] Polymeropoulos MH, Lavedan C, Leroy E (1997). Mutation in the alpha-synuclein gene identified in families with Parkinson’s disease. Science.

[CR72] Prediger RD (2010). Effects of caffeine in Parkinson’s disease: from neuroprotection to the management of motor and non-motor symptoms. J Alzheimers Dis.

[CR73] Qick M (2004). Smoking, nicotine and Parkinson’s disease. Trends Neurosci.

[CR74] Quik M, Jeyarasasingam G (2000). Nicotinic receptors and Parkinson’s disease. Eur J Pharmacol.

[CR75] Rabey JM, Sagi I, Huberman M (2000). Rasagiline mesylate, a new MAO-B inhibitor for the treatment of Parkinson’s disease: a double-blind study as adjunctive therapy to levodopa. Clin Neuropharmacol.

[CR76] Rascol O, Brooks DJ, Melamed E (2005). Rasagiline as an adjunct to levodopa in patients with Parkinson’s disease and motor fluctuations (LARGO, Lasting effect in Adjunct therapy with Rasagiline Given Once daily, study): a randomised, double-blind, parallel-group trial. Lancet.

[CR77] Rockenstein E, Ostroff G, Dikengil F (2018). Combined active humoral and cellular immunization approaches for the treatment of synucleinopathies. J Neurosci.

[CR78] Rojas P, Altagracia M, Kravsov J (1992). Partially protective effect of amantadine in the MPTP model of Parkinson’s disease. Proc West Pharmacol Soc.

[CR79] Ross GW, Abbott RD, Petrovitch H (2000). Association of coffee and caffeine intake with the risk of Parkinson disease. JAMA.

[CR80] Saaksjarvi K, Knekt P, Rissanen H (2008). Prospective study of coffee consumption and risk of Parkinson’s disease. Eur J Clin Nutr.

[CR81] Sas K, Robotka H, Toldi J (2007). Mitochondria, metabolic disturbances, oxidative stress and the kynurenine system, with focus on neurodegenerative disorders. J Neurol Sci.

[CR82] Schapira AH (2008). Progress in neuroprotection in Parkinson’s disease. Eur J Neurol.

[CR83] Schwab RS, Poskanzer DC, England AC (1972). Amantadine in Parkinson’s disease: review of more than 2 years’ experience. JAMA.

[CR84] Seidl SE, Potashkin JA (2011). The promise of neuroprotective agents in Parkinson’s disease. Front Neurol.

[CR85] Seif el Nasr M, Peruche B, Rossberg C (1990). Neuroprotective effect of memantine demonstrated in vivo and in vitro. Eur J Pharmacol.

[CR86] Shults CW, Oakes D, Kieburtz K (2002). Effects of coenzyme Q10 in early Parkinson disease: evidence of slowing of the functional decline. Arch Neurol.

[CR87] Sian-Hülsmann J, Mandel S, Youdim MB (2010). The relevance of iron in the pathogenesis of Parkinson’s disease. J Neurochem.

[CR88] Spencer B, Trinh I, Rockenstein E (2019). Systemic peptide mediated delivery of an siRNA targeting α-syn in the CNS ameliorates the neurodegenerative process in a transgenic model of Lewy body disease. Neurobiol Dis.

[CR89] Stern G (1996). Parkinson’s disease. The apoptosis hypothesis. Adv Neurol.

[CR90] Storch A, Jost WH, Vieregge P (2007). Randomized, double-blind, placebo-controlled trial on symptomatic effects of coenzyme Q(10) in Parkinson disease. Arch Neurol.

[CR91] Tábi T, Vécsei L, Youdim MB (2019). Selegiline: a molecule with innovative potential. J Neural Transm (Vienna).

[CR92] Tan EK, Tan C, Fook-Chong SM (2003). Dose-dependent protective effect of coffee, tea, and smoking in Parkinson’s disease: a study in ethnic Chinese. J Neurol Sci.

[CR93] Tarazi FI, Sahli ZT, Wolny M (2014). Emerging therapies for Parkinson’s disease: from bench to bedside. Pharmacol Ther.

[CR94] Teismann P, Tieu K, Choi DK (2003). Cyclooxygenase-2 is instrumental in Parkinson’s disease neurodegeneration. Proc Natl Acad Sci.

[CR95] Tetrud JW, Langston JW (1989). The effect of deprenyl (selegiline) on the natural history of Parkinson’s disease. Science.

[CR96] The Parkinson Study Group (2004). Levodopa and the progression of Parkinson’s disease. N Engl J Med.

[CR97] The Parkinson Study Group QE3 Investigators (2014). A randomized clinical trial of high dosage Coenzyme Q10 in early Parkinson disease: no evidence of benefit. JAMA Neurol.

[CR98] Tong J, Rathitharan G, Meyer JH (2017). Brain monoamine oxidase B and A in human parkinsonian dopamine deficiency disorders. Brain.

[CR99] Uitti RJ, Rajput AH, Ahlskog JE (1996). Amantadine treatment is an independent predictor of improved survival in Parkinson’s disease. Neurology.

[CR100] Vécsei L, Szalárdy L, Fülöp F (2013). Kynurenines in the CNS: recent advances and new questions. Nat Rev Drug Discov.

[CR101] Verschuur CVM, Suwijn SR, Boel JA (2019). Randomized delayed-start trial of levodopa in Parkinson’s disease. N Engl J Med.

[CR102] Visanji NP, Brooks PL, Hazrati L-N (2013). The prion hypothesis in Parkinson’s disease: Braak to the future. Acta Neuropathol Commun.

[CR103] Weinreb O, Amit T, Riederer P (2011). Neuroprotective profile of the multitarget drug rasagiline in Parkinson’s disease. Int Rev Neurobiol.

[CR104] Whone AL, Watts RL, Stoessl AJ (2003). Slower progression of Parkinson’s disease with ropinirole versus levodopa: The REAL-PET study. Ann Neurol.

[CR105] Youdim MB (2010). Rasagiline in Parkinson’s disease. N Engl J Med.

[CR106] Youdim MB, Gross A, Finberg JP (2001). Rasagiline [N-propargyl-1R(+)-aminoindan], a selective and potent inhibitor of mitochondrial monoamine oxidase B. Br J Pharmacol.

[CR107] Zádori D, Klivényi P, Plangár I (2011). Endogenous neuroprotection in chronic neurodegenerative disorders: with particular regard to the kynurenines. J Cell Mol Med.

[CR108] Zádori D, Klivényi P, Szalárdy L (2012). Mitochondrial disturbances, excitotoxicity, neuroinflammation and kynurenines: novel therapeutic strategies for neurodegenerative disorders. J Neurol Sci.

[CR109] Zádori D, Klivényi P, Toldi J (2012). Kynurenines in Parkinson’s disease: therapeutic perspectives. J Neural Transm.

[CR110] Zádori D, Szalárdy L, Toldi J (2013). Some molecular mechanisms of dopaminergic and glutamatergic dysfunctioning in Parkinson’s disease. J Neural Transm.

[CR111] Zou L, Xu J, Jankovic J (2000). Pramipexole inhibits lipid peroxidation and reduces injury in the substantia nigra induced by the dopaminergic neurotoxin 1-methyl-4-phenyl-1,2,3,6-tetrahydropyridine in C57BL/6 mice. Neurosci Lett.

